# Co-expression network analysis identifies a gene signature as a predictive biomarker for energy metabolism in osteosarcoma

**DOI:** 10.1186/s12935-020-01352-2

**Published:** 2020-06-22

**Authors:** Naiqiang Zhu, Jingyi Hou, Guiyun Ma, Shuai Guo, Chengliang Zhao, Bin Chen

**Affiliations:** 1 Department of Minimally Invasive Spinal Surgery, The Affiliated Hospital of Chengde Medical College, Chengde, 067000 China; 2 Chengde Medical College, Chengde, 067000 China

**Keywords:** Gene signature, Energy metabolism, Least absolute shrinkage and selection operator, Osteosarcoma, Prognosis biomarker, Weighted co-expressed network analysis

## Abstract

**Background:**

Osteosarcoma (OS) is a common malignant bone tumor originating in the interstitial tissues and occurring mostly in adolescents and young adults. Energy metabolism is a prerequisite for cancer cell growth, proliferation, invasion, and metastasis. However, the gene signatures associated with energy metabolism and their underlying molecular mechanisms that drive them are unknown.

**Methods:**

Energy metabolism-related genes were obtained from the TARGET database. We applied the “NFM” algorithm to classify putative signature gene into subtypes based on energy metabolism. Key genes related to progression were identified by weighted co-expression network analysis (WGCNA). Based on least absolute shrinkage and selection operator (LASSO) Cox proportional regression hazards model analyses, a gene signature for the predication of OS progression and prognosis was established. Robustness and estimation evaluations and comparison against other models were used to evaluate the prognostic performance of our model.

**Results:**

Two subtypes associated with energy metabolism was determined using the “NFM” algorithm, and significant modules related to energy metabolism were identified by WGCNA. Gene Ontology (GO) and Kyoto Encyclopedia of Genes and Genomes (KEGG) suggested that the genes in the significant modules were enriched in kinase, immune metabolism processes, and metabolism-related pathways. We constructed a seven-gene signature consisting of SLC18B1, RBMXL1, DOK3, HS3ST2, ATP6V0D1, CCAR1, and C1QTNF1 to be used for OS progression and prognosis. Upregulation of CCAR1, and C1QTNF1 was associated with augmented OS risk, whereas, increases in the expression SCL18B1, RBMXL1, DOK3, HS3ST2, and ATP6VOD1 was correlated with a diminished risk of OS. We confirmed that the seven-gene signature was robust, and was superior to the earlier models evaluated; therefore, it may be used for timely OS diagnosis, treatment, and prognosis.

**Conclusions:**

The seven-gene signature related to OS energy metabolism developed here could be used in the early diagnosis, treatment, and prognosis of OS.

## Introduction

Osteosarcoma (OS) is one of the most common malignant bone tumors. It appears mainly in adolescents and young adults (overall incidence: 0.3–0.4/100,000 individuals/year) [1, 2]. As with other sarcomas, OS originates from the interstitial tissues. It is characterized by the formation of osteoid tissue and osteoid- and spindle-shaped stromal cells in immature bones [3, 4]. The main clinical treatments for patients with OS include local surgery, chemotherapy, and radiotherapy. Substantial improvements have been made in the clinical response and survival rate of OS [5]. About 70% of all patients with OS can be cured [6]. However, OS prognosis remains poor especially among patients with metastatic disease or tumor recurrence. In these cases, the overall survival rate is only ~ 20% [7]. Despite advances in surgical technique and targeted therapy, optimal treatment outcomes in OS are still negatively impacted by tumor immunity, infection, complications, and low survival rates. Thus, new predictive and prognostic approaches are urgently needed to improve survival in patients with OS.

High-throughput technology, gene microarray chips, and large-scale RNA-seq transcriptome data have been widely used to identify prognostic genes for various cancers, elucidate oncogenic mechanisms, and improve cancer treatment [8, 9]. Energy metabolism is an important marker of cancer cell metastasis and invasion [10]. It enables tumor cells to generate ATP, maintain the redox balance, and sustain the macromolecular biosynthetic processed required for cell growth, proliferation, and migration [11]. Recent empirical evidence has demonstrated that there is a metabolic symbiosis between glycolysis and oxidative phosphorylation (OXPHOS) in OS [12]. Lactate and pyruvate formed in glycolysis may be transferred to the TCA cycle and used as intermediate substrates for ATP generation [13, 14]. OS cells may also utilize the ketones released by adjacent cells during free fatty acid catabolism to liberate metabolic energy [15]. Thus, a new energy metabolism-related gene signature could be invaluable in the prediction of OS metastasis and invasion.

**Fig. 1:**
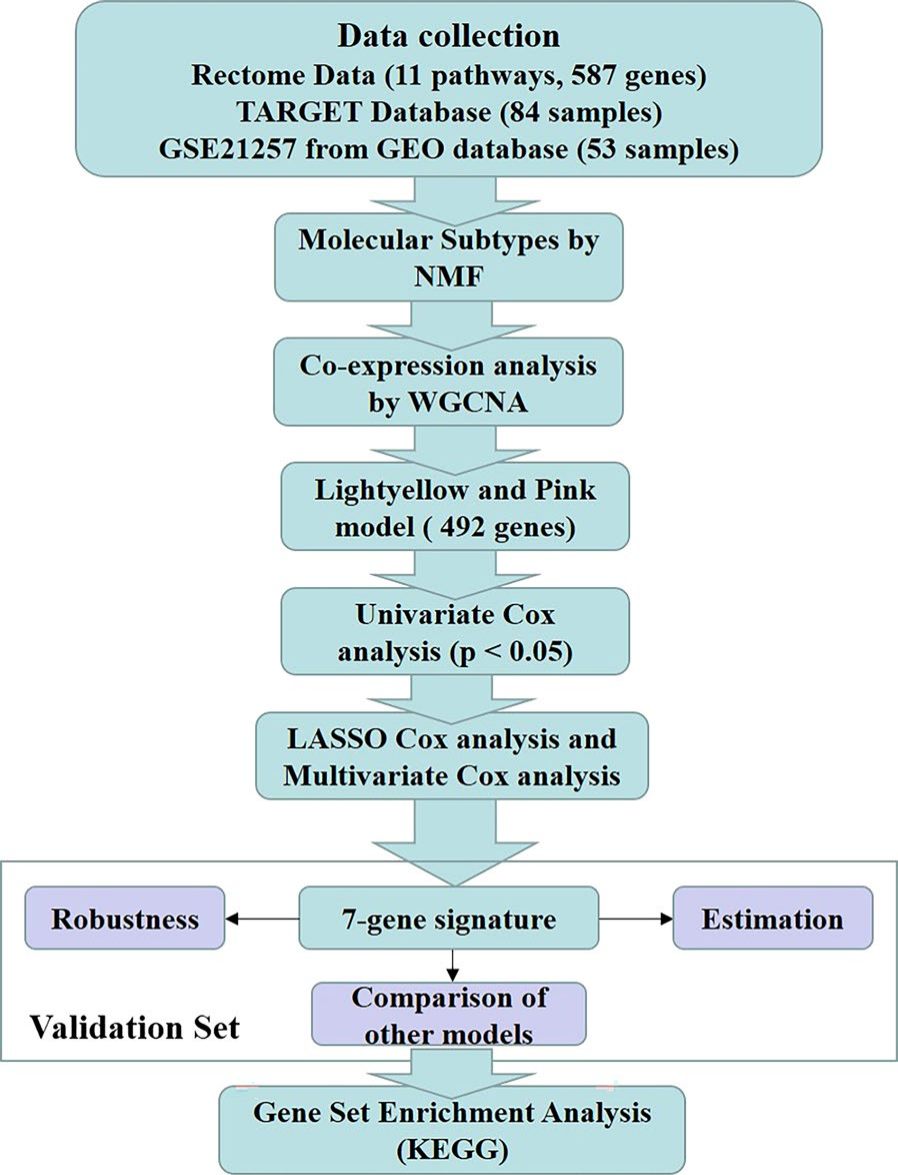
Flow diagram of data preparation, processing, analysis, and validation in the present study

Weighted gene co-expression network analysis (WGCNA) is a systematic biological method that delineates correlations between genes and clinical traits [16]. It identifies highly correlated genes to investigate potential biological functions [17, 18]. Here, a new seven-gene signature associated with OS energy metabolism was established via a WGCNA algorithm and a least absolute shrinkage and selection operator (LASSO) Cox regression. This seven-gene signature was validated and confirmed to be superior to other predictive models of the same type. It served as a biomarker that was significantly associated with OS metastasis. Our findings may advance the study of OS prognosis, as they revealed that energy metabolism is a potential therapeutic control point in this disease. The research path taken in the present study is outlined in Fig. 1.

## Results

### Identification of the energy metabolism molecular subtypes in OS

**Fig. 2:**
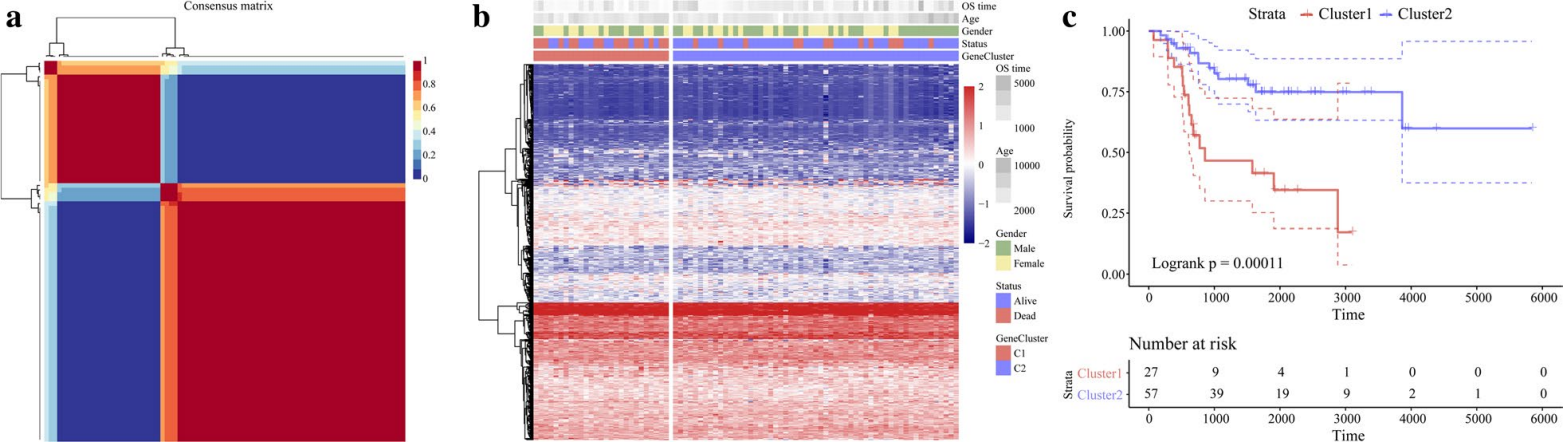
Molecular subtype classification according to energy metabolism in TARGET Database. **a** Consensus map of NMF clustering. **b** Heatmap of energy metabolism-related gene expression by molecular subtype. **c** Prognosis survival curve by molecular subtype

**Fig. 3:**
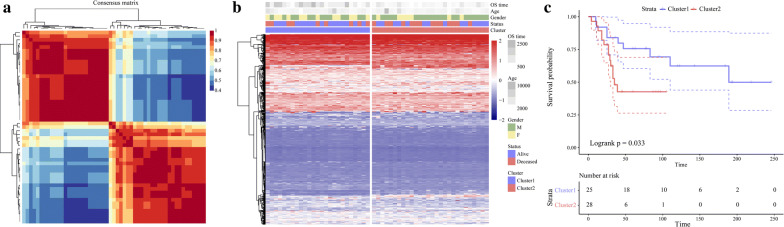
Molecular subtype classification according to energy metabolism in GSE21257. **a** Consensus map of NMF clustering. **b** Heatmap of energy metabolism-related gene expression by molecular subtype. **c** Prognosis survival curve of molecular subtype

The “NFM” algorithm [19] was used to identify the molecular subtypes of energy metabolism in OS samples with 587 identified energy metabolism-related genes [20]. As shown in Fig. 2a, the energy metabolism molecular subtypes of OS were detected in the TARGET database using cophenetic, dispersion and silhouette algorithm indicators. The expression levels of the energy metabolism-related genes in the two molecular subtypes are shown in Fig. 2b. Patient mortality was significantly higher in the C1 group than the C2 group. We also determined that the prognosis for C1 was worse than that for C2. Figure 2c shows significant differences between these molecular subtypes (log-rank *P* = 0.032). Using the same parametric analysis as for the TARGET data, we ran the “NMF” algorithm on the GEO dataset GSE21257 and found similarities between them. Both were partitioned into two groups. As shown in Fig. 3, there were significant differences between subtypes in terms of prognosis (log-rank *P* = 0.033).

### Detection of significant modules in the molecular subtypes

**Fig. 4:**
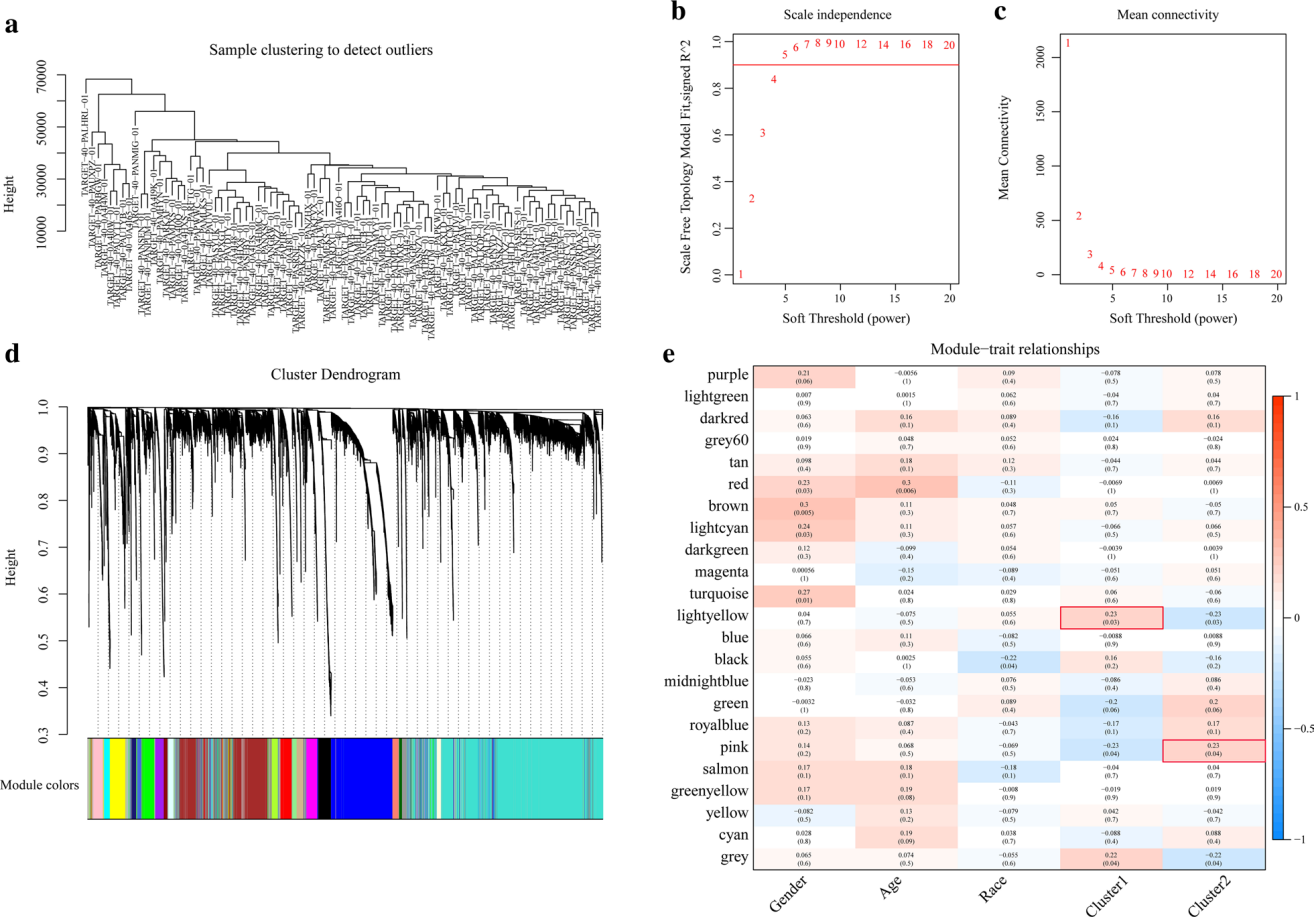
Co-expression network analysis. **a** Sample clustering analysis. **b**, **c** Analysis of network topology showing that it met the scale-free topology threshold of 0.9 when β = 5. **d** Clustering dendrogram of genes based on topological overlap. **e** Heatmap displaying correlations and significant differences between gene modules and clinical phenotypes

WGCNA was performed on the genes in the TARGET database to screen for modules that were significantly associated with the energy metabolism molecular subtypes in OS [21] (Fig. 4). There were no outliers in the sample clustering (Fig. 4a). A value of five was the lowest power for the 0.9 scale-free topology network index. It was screened out in order to plot a hierarchical clustering tree (Fig. 4b, c). Similar clusters were merged into new modules using the following settings: height = 0.5, deepSplit = 2, and minModuleSize = 80. Twenty-three modules with similar connected gene patterns were obtained (Fig. 4d). As shown in Fig. 4e, correlations of each module with patient gender, age, ethnicity, and Clusters1 and 2 were analyzed. The strongest association was found between the lightyellow module and Cluster 1, and between the pink module and Cluster 2. Therefore, the lightyellow (140 genes) and pink (352 genes) modules were selected for the subsequent analyses (Additional file 1: Table S1 and Additional file 2: Table S2).

### Gene Ontology (GO) and pathway enrichment analysis

**Fig. 5:**
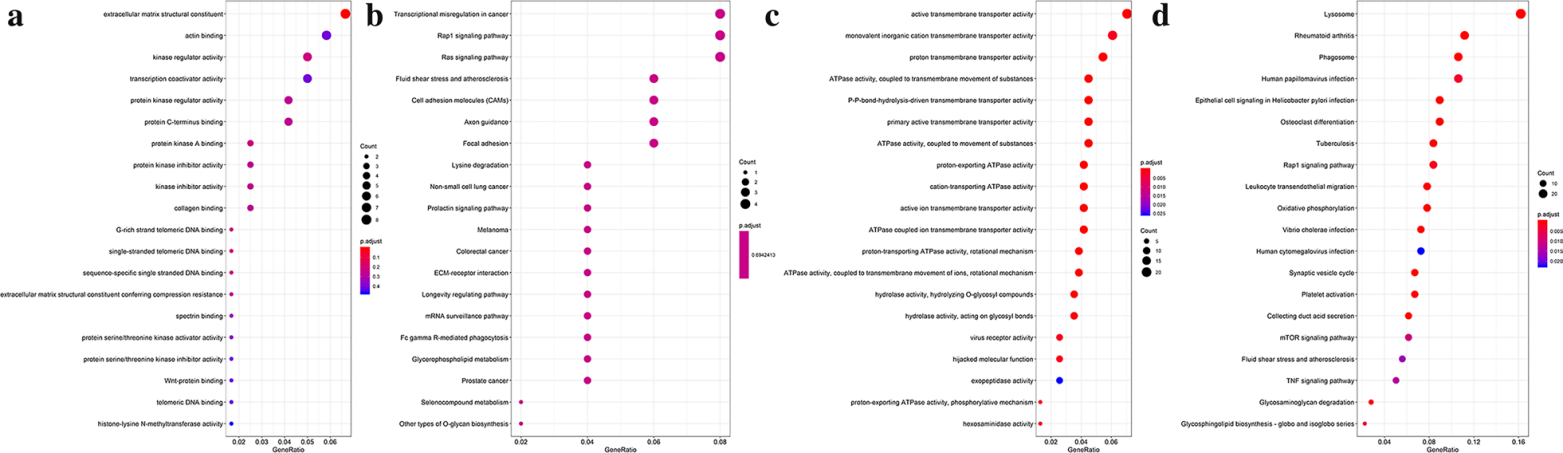
Functional enrichment analysis of lightyellow and pink modules. **a** Top 20 GO terms in lightyellow module. **b** Top 20 KEGG terms in lightyellow module. **c** Top 20 GO terms in pink module. **d** Top 20 KEGG terms in pink module

GO and KEGG functional enrichment analyses of the genes in the lightyellow and pink modules were performed using the “ClusterProfiler” package in R (Additional file 3: Table S3, Additional file 4: Table S4, Additional file 5: Table S5, Additional file 6: Table S6). As shown in Fig. 5a, the genes in the lightyellow module were enriched in 293 GO terms closely related to kinase activity. They were also enriched in the Ras and Rap1 signaling pathways and ECM-receptor interaction (Fig. 5b). The genes in the pink module were enriched in 567 GO terms and 263 KEGG terms. The top 20 GO terms (Fig. 5c) were related mainly to immune metabolism processes such as neutrophil-mediated immunity, and ATP hydrolysis-coupled proton transport. The KEGG analysis suggested that the top 20 significantly enriched pathways were related to tumorigenesis, including the mTOR and TNF signaling pathways (Fig. 5d).

### LASSO Cox regression and energy metabolism signature

**Fig. 6:**
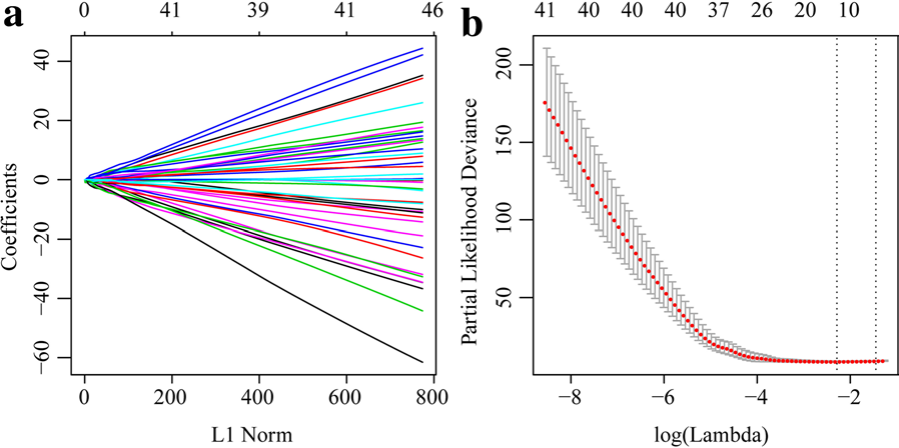
Constructing seven-gene-based classifier by LASSO Cox regression model. **a** Trajectory of each independent variable. Horizontal axis represents log of independent variable λ. Vertical axis represents coefficient of independent variable. **b** Three fold cross-validation of tuning parameter in LASSO model

A univariate Cox proportional hazard regression model was applied to screen for significant differences in the final prognoses. Sixty-five significant genes were obtained of which the top 20 are listed in Table 1. LASSO Cox regression was performed on all 65 significant OS-related genes. As shown in Fig. 6a, the trajectory of each independent variable was analyzed. As λ increased, the number of independent coefficients tended to decline towards zero. A threefold cross-validation was run to build the model and the confidence interval under each λ was analyzed. The model was optimal when λ = 0.101608. Thirteen genes with λ = 0.101608 were chosen as the final targets (Fig. 6b) and subjected to multivariate Cox survival analysis. Seven genes with the lowest AUC (156.28) were retained and integrated into the final model (Fig. 6c; Table 2). The RiskScore was calculated for the seven-gene signature as follows:

$$ \begin{aligned} {\text{RiskScore7}} & = - 1. 2 5 1 4\times {\text{expSLC18B1}} - 1. 4 7 6\times {\text{expRBMXL1}} \\ & \quad + 1. 5 1 4 5\times {\text{expC1QTNF1}} - 1. 3 60 6\times {\text{expDOK3}} \\ & \quad + 1. 1 6 3 8\times {\text{expCCAR1}} - 0. 7 1 2 7\times {\text{expHS3ST2}} - 2. 1 8 6\times {\text{expATP6V}}0{\text{D1}} .\\ \end{aligned} $$

**Table 1:** Top 20 significant lncRNA screened by univariate Cox regression analysis

Symbol	P-value	HR	Low 95% CI	High 95% CI
NUBP1	1.32E−05	0.256	0.138	0.472
PDZD11	0.00023	0.274	0.138	0.546
ATP6V0D1	0.00037	0.380	0.223	0.648
NTAN1	0.00064	0.391	0.228	0.670
TMEM8A	0.00136	0.372	0.203	0.681
FOLR2	0.00176	0.664	0.513	0.858
GRN	0.00247	0.565	0.390	0.818
BLVRA	0.00347	0.429	0.243	0.757
NPC2	0.00387	0.582	0.403	0.840
RBMXL1	0.00398	0.372	0.190	0.729
DOK3	0.00414	0.532	0.346	0.819
APBB1IP	0.00427	0.615	0.440	0.858
GPX1	0.00497	0.547	0.359	0.833
FCER1G	0.00673	0.684	0.519	0.900
TWF2	0.00675	0.459	0.261	0.806
PLEKHO2	0.00729	0.464	0.265	0.813
CHCHD10	0.00817	0.596	0.406	0.875
HS3ST2	0.00819	0.541	0.343	0.853
TAGLN	0.00884	0.650	0.470	0.897
SELPLG	0.00933	0.635	0.451	0.894

**Table 2:** Information for seven-gene signature screened by LASSO Cox regression

Symbol	Coef	P value	HR	Low 95% CI	High 95% CI
SLC18B1	− 1.2514	0.13982	0.2861	0.05433	1.5065
RBMXL1	− 1.476	0.09257	0.2286	0.04093	1.2762
C1QTNF1	1.5145	4.08E−05	4.5473	2.20564	9.3749
DOK3	− 1.3606	0.03348	0.2565	0.07318	0.8991
CCAR1	1.1638	0.1236	3.2021	0.72794	14.0852
HS3ST2	− 0.7127	0.13179	0.4903	0.19404	1.2389
ATP6V0D1	− 2.186	0.00587	0.1124	0.02373	0.5322

**Fig. 7:**
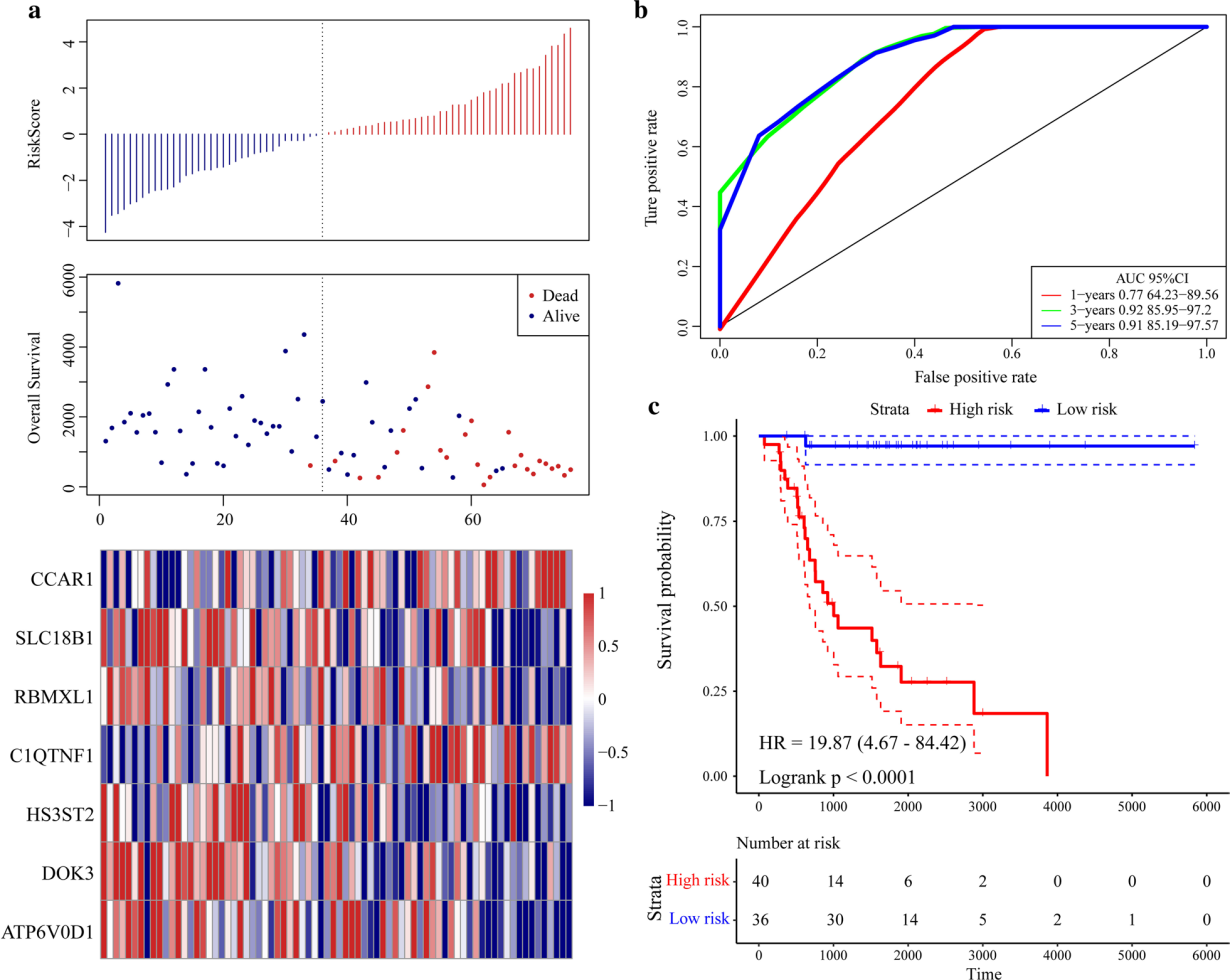
Evaluation of robustness of seven-gene signature in TARGET training datasets. **a** RiskScore, survival time and status, and expression of seven-gene signature. **b** ROC curve of seven-gene signature. **c** K–M prognosis curve of seven-gene signature

The scoring formula for each sample is the sum of the aforementioned gene expression value multiplied by the ordinal. We set the optimal threshold calculated from the 5-year AUC as the classification effect in the TARGET training set. As shown in Fig. 7a, the death sample survival time significantly decreased with increasing patient RiskScore. Most of the individuals who died were in the high-risk group. The expression levels of CCAR1 and C1QTNF1 increased with risk value. Thus, upregulation of either of these genes is associated with high risk of death. In contrast, the expression levels of SCL18B1, RBMXL1, DOK3, HS3ST2, and ATP6VOD1 decreased with increasing risk value. Therefore, upregulation of these genes is correlated with low risk and all five of them are protective. Figure 7b shows the ROC curve for all seven genes. The AUC was > 0.74. As shown in Fig. 7c, 36 patients were classified into the low-risk group and 40 were assigned to the high-risk group. There was a significant difference between these two groups (log-rank *P* < 0.001; HR = 19.87).

### Validation of gene signature robustness

**Fig. 8:**
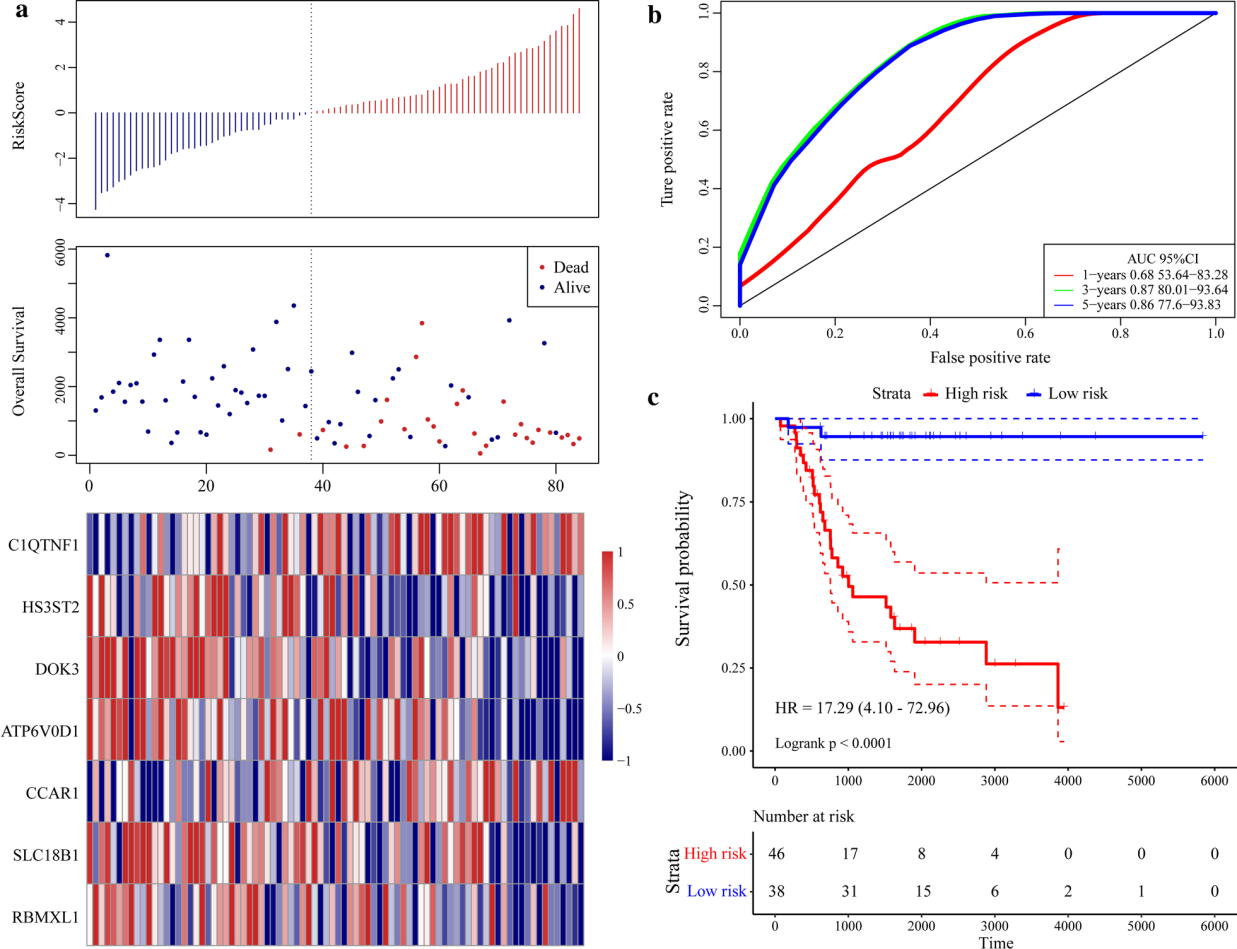
Evaluation of robustness of seven-gene signature in TARGET validation datasets. **a** RiskScore, survival time and status, and the expression of seven-gene signature. **b** ROC curve of seven-gene signature. **c** K–M prognosis curve of seven-gene signature

**Fig. 9:**
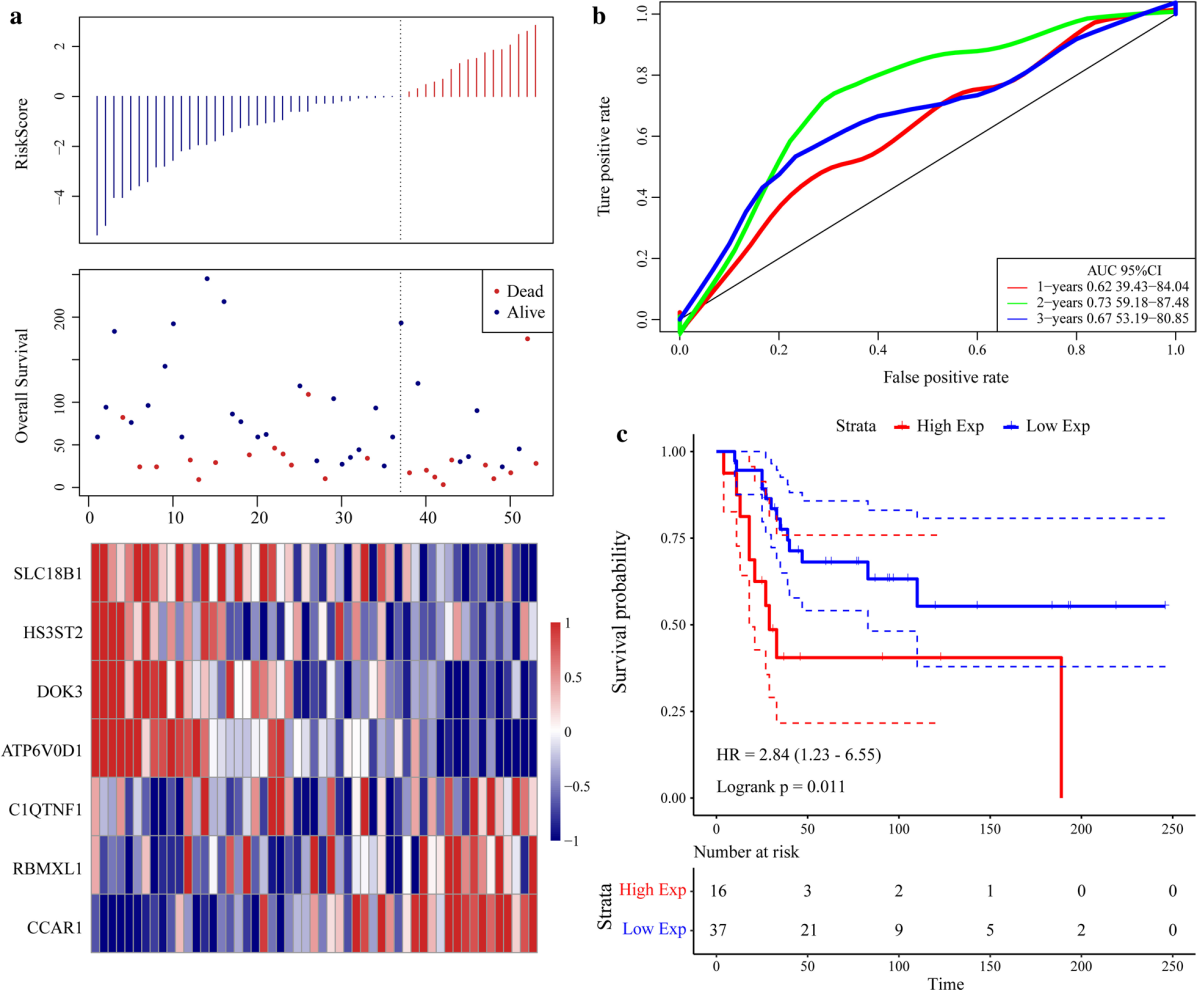
Evaluation of robustness of seven-gene signature in GEO validation. **a** RiskScore, survival time and status, and the expression of seven-gene signature. **b** ROC curve of seven-gene signature. **c** K–M prognosis curve of seven-gene signature

**Fig. 10:**
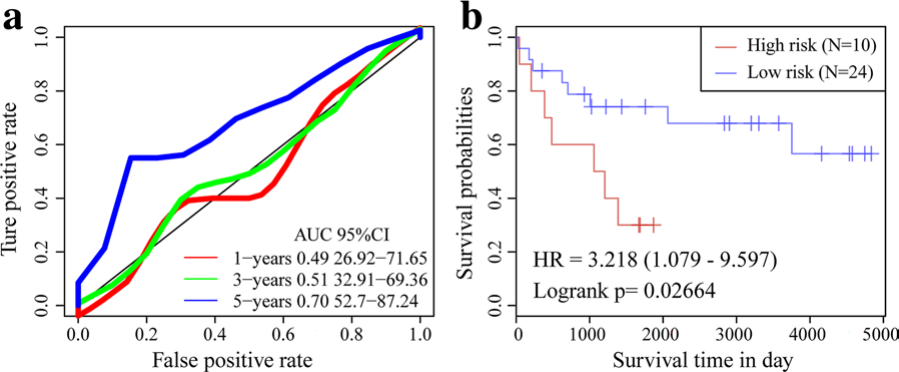
Evaluation of robustness of seven-gene signature in GSE16091. **a** ROC curve of seven-gene signature. **b** K–M prognosis curve of seven-gene signature

To validate the robustness of the gene signature, we calculated the RiskScore for the expression level of each sample in the validation set. The RiskScore distribution is shown in Fig. 8a. The expression level of the seven-gene signature increased with risk value. These findings are consistent with the training set in which the expression levels of CCAR1 and C1QTNF1 also increased with risk value. Therefore, high expression and high risk were associated with these two genes and both were risk factors. In contrast, the expression levels of SLC18B1, RBMXL1, DOK3, HS3ST2, and ATP6V0D1 decreased with increasing risk value. There was a correlation between high expression and low risk for these five genes and all of them were protective factors. ROC analysis of the prognostic RiskScore classification was performed using the “timeROC” package in R. As shown in Fig. 8b, the model had a high AUC value (0.87). Figure 8c suggested that according to classification of all TARGET sets, 38 patients were scored as low-risk and 46 patients were rated as high-risk. There were significant differences between these risk groups (log-rank P < 0.0001; HR = 17.92). The external GEO dataset GSE21257 was analyzed by the same method as above to verify the model robustness. It generated the same results as the TARGET validation and training sets, namely, CCAR1 and C1QTNF1 were risk factors, whereas SLC18B1, RBMXL1, DOK3, HS3ST2, and ATP6V0D1 were protective factors (Fig. 9a). Figure 9b depicts that the model had a high AUC value (0.73). Based on the GSE21257 data, 37 patients were classified as low-risk and 16 patients were classified as high-risk, and again the difference between the groups was significant (log-rank *P* = 0.011; HR = 2.84) (Fig. 9c). The foregoing results indicate that the seven-gene signature was highly robust. In addition, the external dataset GSE16091 validated results suggested this seven-gene signature had a high AUC value (0.7); 10 patients and 24 patients were classified into low-risk and high-risk groups, respectively (log-rank *P* = 0.02664; HR = 3.218) (Fig. 10).

### Gene signature model evaluation

**Fig. 11:**
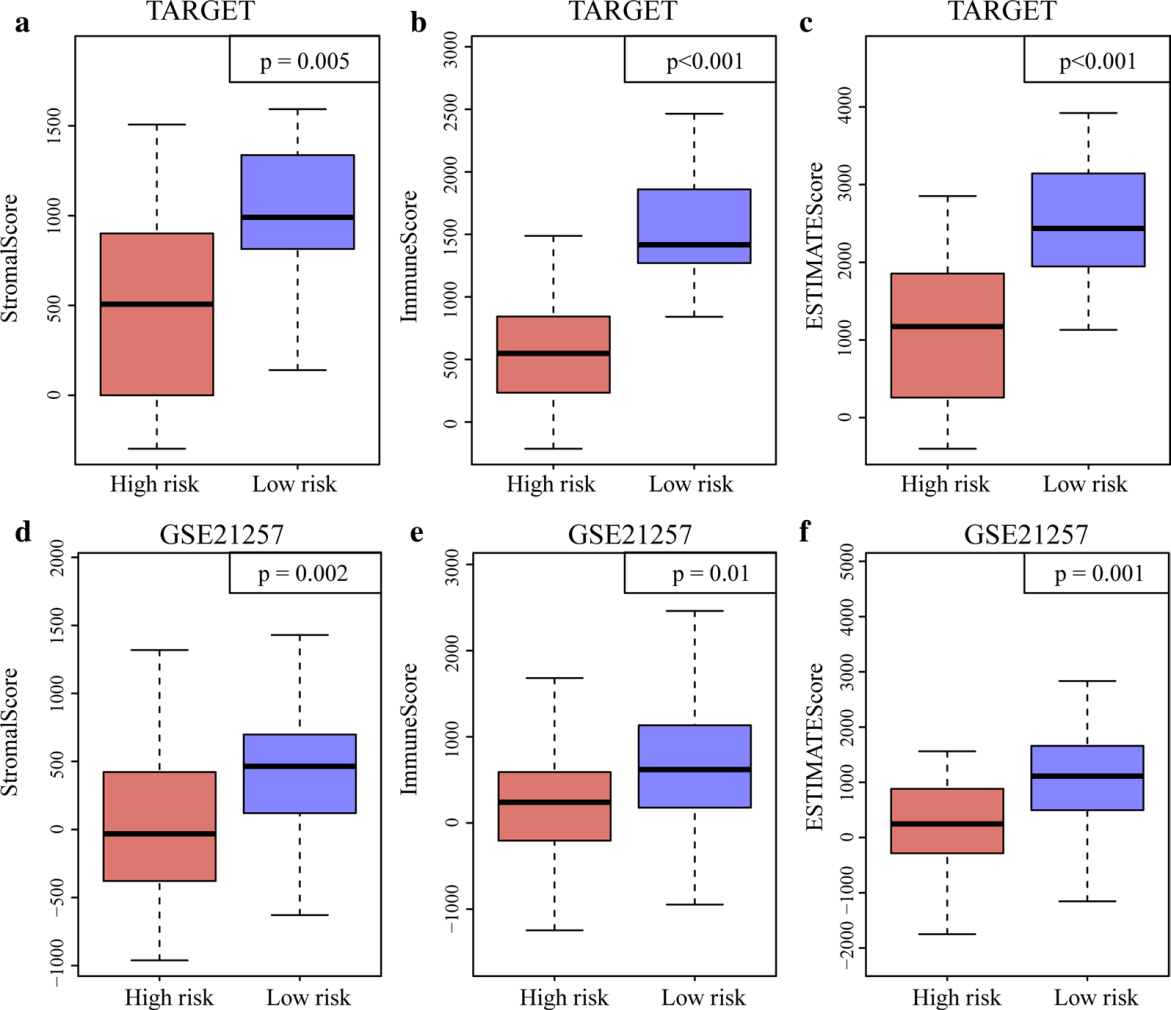
Estimation of seven-gene signature. **a** Distributions of StromalScore in high and low-risk groups in TARGET training dataset. **b** Distributions of ImmuneScore in high and low-risk groups in TARGET training dataset. **c** Distributions of ESTIMATEScore in high and low-risk groups in TARGET training dataset. **d** Distributions of StromalScore in high and low-risk groups in GSE21257 training dataset. **e** Distributions of ImmuneScore in high and low-risk groups in GSE21257 training dataset. **f** Distributions of the ESTIMATEScore in high and low-risk groups in GSE21257 training dataset

To identify the relationship between the RiskScore for the seven-gene signature and the immune and matrix scores, the ImmuneScore, StromalScore, and tumor purity were calculated separately for each sample [22]. There were significant differences between the high-risk and low-risk samples in the TARGET training set in terms of ImmuneScore, StromalScore, and tumor purity. Similar results were obtained for the GSE21257 dataset (Fig. 11). To confirm the independence of the seven-gene signature model in a clinical setting, we systematically analyzed the clinical information in the TARGET, TARGET training, and GSE21257 validation datasets including age, gender, metastasis, and seven-gene signature model grouping information (Table 3). One-way Cox regression analysis of the TARGET training set revealed that the high-risk group and metastasis were significantly associated with survival. The corresponding multivariate Cox regression analysis revealed that only the high-risk group (HR = 28.89; 95% CI 6.25–133.4; *P* = 1.63E^−05^), age, and metastasis were significantly associated with survival. A one-way Cox regression analysis of the TARGET set demonstrated that the high-risk group and metastasis were significantly associated with survival. Similarly, the multivariate Cox regression analysis showed that only the high-risk group (HR = 21.07; 95% CI 4.87–91.21; *P *= 4.5E^−05^) and metastasis were significantly associated with survival. One-way and multivariate Cox regression analysis of the GSE21257 validation set indicated that the high-risk group was significantly associated with survival. The aforementioned analyses confirmed that the seven-gene signature is an independent standalone prognostic indicator with good predictive performance and clinical application utility.

**Table 3:** Univariate and multivariate Cox regression analyses, prognosis-related clinical factors, and clinical independence

Variables	Univariate analysis			Multivariable analysis		
	HR	95% CI of HR	P value	HR	95% CI of HR	P value
TARGET training datasets						
7-gene risk score						
Risk score (high/low)	19.87	4.67–84.42	3.11E−04	28.89	6.25–133.4	1.63E−05
Age	0.99	0.90–1.09	0.83	1.12	1.00–1.24	0.048
Gender (male/female)	0.57	0.25–1.27	0.17	0.57	0.24–1.33	0.19
Metastatic vs non-metastatic	4.18	1.92–9.10	0.0003	5.34	2.21–12.95	0.0002
TARGET datasets						
7-gene risk score						
Risk score (high/low)	17.29	4.10–72.96	1.04E−04	21.07	4.87–91.21	4.54E−05
Age	0.99	0.91–1.07	0.81	1.08	0.98––1.19	0.098
Gender (male/female)	0.76	0.36–1.60	0.47	0.76	0.35–1.64	0.48
Metastatic vs Non-metastatic	4.74	2.27–9.89	3.42E−05	5.69	2.54–12.76	2.36E−05
GSE21257 validation datasets						
7-gene risk score						
Risk score (high/low)	2.84	1.23–6.55	0.014	2.76	1.18–6.46	0.0186
Age	1.0007	0.99–1.003	0.602	1	0.99–1.003	0.787
Gender (male/female)	1.4	0.58–3.34	0.44	1.39	0.56–3.41	0.468

### Gene set enrichment analysis (GSEA)

GSEA was performed on the significantly enriched pathways in the high- and low-risk groups in the TARGET set. The enriched pathway selection threshold was *P* < 0.05, and significantly enriched pathways are listed in Table 4. The high-risk group was mainly associated with metabolic pathways including NITROGEN METABOLISM and LINOLEIC ACID METABOLISM, whereas the low-risk group was enriched mainly in receptor-related pathways, such as B CELL RECEPTOR SIGNALING, CHEMOKINE SIGNALING, and TOLL LIKE RECEPTOR SIGNALING.

**Table 4:** KEGG pathways significantly enriched in high and low-risk groups

Name	Size	ES	NES	NOM	FDR	FWER
				P-val	P-val	P-val
KEGG_NITROGEN_METABOLISM	23	0.497	1.539	0.037	0.470	0.8
KEGG_LINOLEIC_ACID_METABOL ISM	29	0.573	1.572	0.044	0.745	0.753
KEGG_PEROXISOME	78	− 0.483	− 1.871	0.002	0.151	0.176
KEGG_CHEMOKINE_SIGNALING_ PATHWAY	185	− 0.443	− 1.785	0.006	0.144	0.32
KEGG_FC_GAMMA_R_MEDIATED_GOCYTOSIS	95	− 0.479	− 1.821	0.006	0.119	0.244
KEGG_LEUKOCYTE_TRANSENDO THELIAL_MIGRATION	115	− 0.475	− 1.850	0.008	0.109	0.195
KEGG_PATHOGENIC_ESCHERICHI A_COLI_INFECTION	55	− 0.521	− 1.883	0.010	0.200	0.162
KEGG_LYSOSOME	119	− 0.568	− 1.956	0.010	0.167	0.081
KEGG_LEISHMANIA_INFECTION	69	− 0.608	− 1.866	0.012	0.118	0.178
KEGG_VIRAL_MYOCARDITIS	68	− 0.557	− 1.757	0.012	0.166	0.384
KEGG_ENDOCYTOSIS	178	− 0.378	− 1.581	0.017	0.317	0.729
KEGG_SNARE_INTERACTIONS_INICULAR_TRANSPORT	38	− 0.496	− 1.630	0.022	0.295	0.631
KEGG_GLUTATHIONE_METABOLI SM	48	− 0.455	− 1.648	0.025	0.283	0.605
KEGG_TOLL_LIKE_RECEPTOR_SI GNALING_PATHWAY	101	− 0.411	− 1.567	0.032	0.329	0.752
KEGG_ANTIGEN_PROCESSING_A ND_PRESENTATION	79	− 0.518	− 1.661	0.033	0.282	0.575
KEGG_COMPLEMENT_AND_COAG ULATION_CASCADES	69	− 0.457	− 1.590	0.037	0.318	0.715
KEGG_B_CELL_RECEPTOR_SIGNA LING_PATHWAY	74	− 0.427	− 1.591	0.037	0.339	0.714
KEGG_NOD_LIKE_RECEPTOR_SIG NALING_PATHWAY	61	− 0.454	− 1.598	0.037	0.351	0.703
G_ACUTE_MYELOID_LEUKEMA	57	− 0.445	− 1.548	0.041	0.316	0.782

### Comparative study of other risk models

**Fig. 12:**
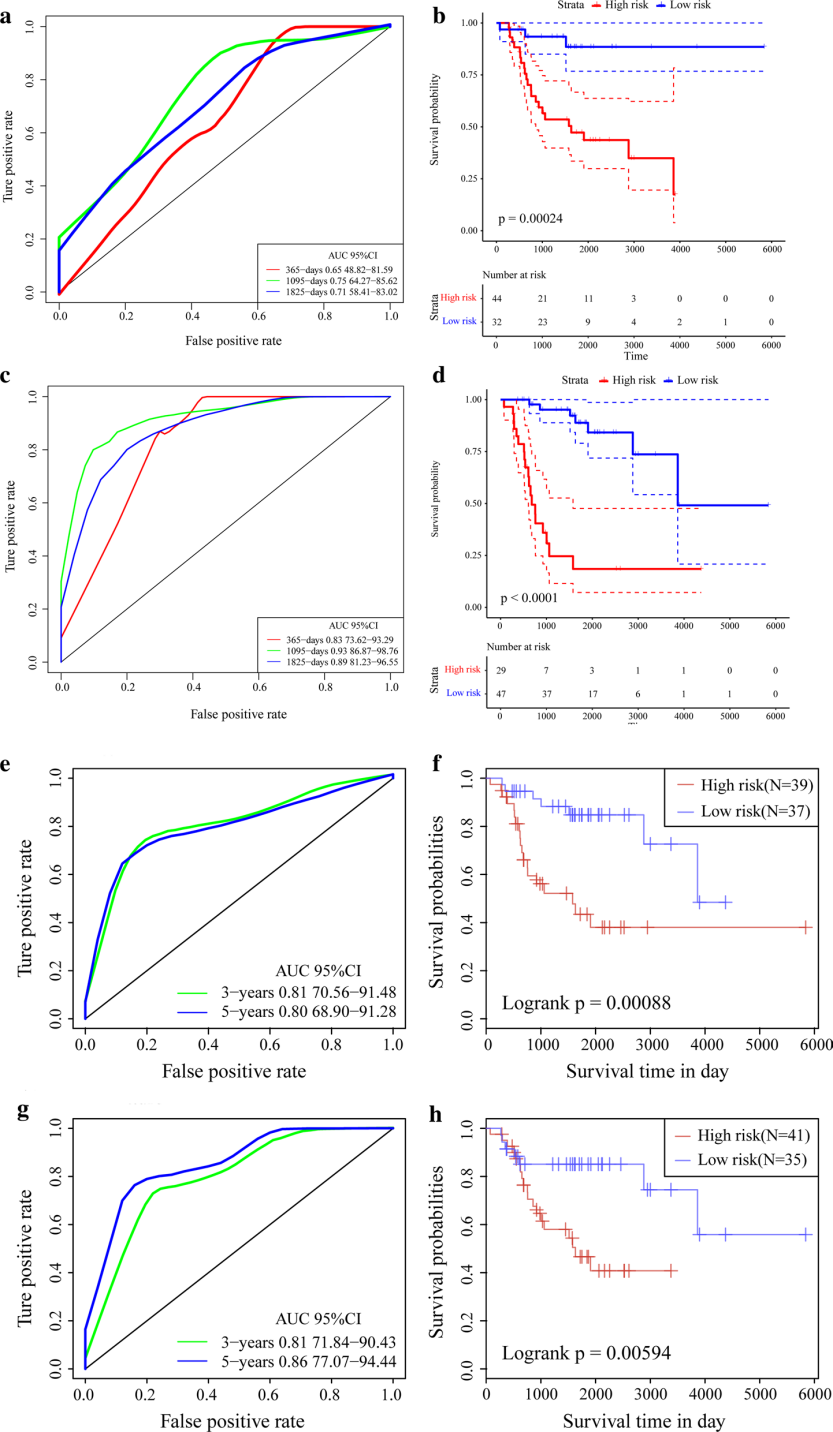
Comparative analysis of other models. **a** AUC curve of eight-gene signature (PMID: 31333788) in TARGET training dataset. **b** K–M curve of eight-gene signature (PMID: 31333788) in TARGET training dataset. **c** AUC curve of four-pseudogene signature (PMID: 31146489) in TARGET training dataset. **d** K–M curve of four-pseudogene signature (PMID: 31146489) in TARGET training dataset. **e** AUC curve of ten-gene signature (PMID: 31090103) in TARGET training dataset. **f** K–M curve of ten-gene signature (PMID: 31090103) in TARGET training dataset. **g** AUC curve of nineteen-pseudogene signature (PMID: 30604867) in TARGET training dataset. **h** K–M curve of 19-pseudogene signature (PMID: 30604867) in TARGET training dataset

We compared published OS-related models with that used in the present study. We determined the differences between the high- and low-risk groups in terms of OS prognosis. As shown in Fig. 12a, b, the ROC and K–M curves (PMID: 31333788) indicated that the 3-year AUC was 0.75 (*P* = 0.00024). Moreover, the OS prognosis for the four-pseudogene signature (PMID: 31146489) demonstrated that the 3-year AUC value was 0.93 (*P* < 0.0001) (Fig. 11c, d). In addition, the ROC and K–M curves in PMID: 31090103 and PMID: 30604867 suggested the 3-year AUC were 0.81(*P *= 0.00088) and 0.81 (*P* = 0.00594), respectively. Comprehensive comparative studies disclosed that the seven-gene signature in the present study was superior to the eight-gene signature (PMID: 31333788) and had predictive power similar to that of the four-pseudogene signature (PMID: 31146489). Thus, the model constructed in the present study has superior performance to the previously published models against which it was compared.

## Discussion

OS is a bone tumor that occurs mainly in teenagers and young adults. It originates from primitive transformed mesenchymal cells. Energy metabolism is an important indicator of osteosarcoma cell proliferation, metastasis, and invasion. In the present study, a comprehensive bioinformatics analysis identified a seven-gene signature associated with OS energy metabolism. This gene signature was significantly associated with OS progression and prognosis.

The “NMF” algorithm was run to partition the samples into glycolysis and OXPHOS energy metabolism categories. Recent investigations found that the relationship between glycolysis and OXPHOS is cooperative and competitive [23]. As OXPHOS is attenuated, glycolysis may be enhanced to increases energy generation. When OXPHOS function is in equilibrium, it regulates glycolysis and maintains the energy balance [24]. Fantin et al. [25] reported that when LDH-A was inhibited in cancer cells, OXPHOS was enhanced to compensate for glycolysis suppression and ATP reduction. Pacheco-Velazquez et al. [26] proposed that MCF-7 cells are equally dependent on OXPHOS and glycolysis for ATP generation.

WGCNA is a systematic biological algorithm revealing the associations between genes and clinical phenotypes. It has been widely used to screen for diagnostic and prognostic biomarkers of Alzheimer’s disease, breast cancer, osteoarthritis, and Dupuytren’s contracture. In the present study, WGCNA identified 23 modules of which the lightyellow and pink were highly associated with molecular subtypes related to OS energy metabolism. A GO analysis disclosed that the genes in these two modules were enriched mainly for protein kinase A, DNA metabolism, and Wnt protein-binding pathways and the transcription coactivator, kinase regulator, ATPase, and proton transmembrane transport activity pathways. Kinases catalyze the transfer of phosphate groups from high-energy, phosphate-donating molecules to specific substrates. Takahashi et al. [27] used siRNA and the small molecule inhibitor CX-4945 to show that upregulation of casein kinase 2 (CK2) was important for the growth of human osteosarcoma cells. Zhu et al. [28] reported that the checkpoint kinase inhibitor AZD7762 promoted apoptosis and mitotic catastrophe in osteosarcoma cells. ATPase decomposes ATP into ADP and free phosphate ion and releases energy. Meszaros et al. [29] found that Ca^2+^-ATPase inhibitors suppress ATP- and thrombin-Ca^2+^ in OS cells. KEGG enrichment analysis showed that the genes in the lightyellow and pink modules participated in osteoclast differentiation, the mTOR, TNF, and Ras signaling pathways, and the focal adhesion-related metabolic pathways. Osteoclast differentiation is involved in bone formation and originates in mesenchymal stem cells. Gobin et al. [30] suggested that the PIDK inhibitor BYL719 inhibited and promoted osteoclast differentiation. The mTOR pathway is the entry point for OS treatment. It regulates cell growth, increases cell proliferation, and suppresses autophagy [31]. Perry et al. [32] used complementary genomics to identify OS-related genomic events. They found that inhibition of the mTOR pathway could be exploited for OS therapy. Focal adhesion is a key regulator of multi-cellular signaling pathways in cell proliferation, cycle regression, migration, apoptosis, and survival [33]. Hu et al. [34] assessed the effects of the small-molecule focal adhesion inhibitor PF562271 on OS cells and showed that it diminished OS cell volume, weight, and angiogenesis and concluded that inhibition of focal adhesion could therefore be a target for OS treatment.

As the lightyellow and pink modules were screened by WGCNA, a systematic LASSO Cox regression estimation was performed, which compresses the coefficients and conserves the original data. The LASSO Cox regression screened seven genes that were then used to construct a seven-gene signature with the lowest AUC value. This seven-gene signature was validated by robustness and estimation evaluations, and by comparing it to other models from independent external datasets with the TARGET training dataset. These analyses revealed the clinical significance of the signature in CCAR1, and C1QTNF1 (risk factor), and in SLC18B1, RBMXL1, DOK3, HS3ST2, and ATP6V0D1 (protective factors). Metastasis and invasion information for clinical biopsies or surgical samples may be obtained by quantifying the expression level of each gene in this seven-gene signature. Genes within this signature may participate in cancer development and progression. *CCAR1* (cell division cycle and apoptosis regulator protein 1) is an intermediate in regulatory transduction. It is involved in transcriptional regulation, apoptosis, autophagy, and cell progression and/or proliferation. CCAR1 is a biomarker of hepatocellular carcinoma [35]. *RBMXL1* (RNA binding motif protein) encodes a splicing protein that suppresses tumor proliferation, and promotes apoptosis in gastric and breast cancer [36, 37]. *DOK3* (downstream of tyrosine kinase 3) is an adaptor protein that plays a vital role in the negative feedback regulation of PTK-mediated signaling loops and suppresses the cellular proliferation [38]. *HS3ST2* (heparan sulfate-glucosamine 3-sulfotransferase 2) encodes an enzyme that [39] participates in cell proliferation, apoptosis, autophagy, and other processes associated with cancer [40]. ATP6V0D1 (V-type proton ATPase subunit d1) acidifies various intracellular compartments in cancer cells and generates transport process energy [41]. Han et al. [42] suggested that C1QTNF1 (complement C1q tumor necrosis factor-related protein 1) acts on the miR-221-3p/SOCS3 axis to modulate the JAK/STAT signaling pathway and alter HCC cell behavior and tumor proliferation. SLC18B1 (MFS-type transporter SLC18B1) has not yet been functionally linked to cancer, but we suggest it could be a viable target for OS treatment.

In conclusion, we used comprehensive bioinformatics techniques to define a novel and effective biomarker for the prediction of the clinical progression, development, invasion, and metastasis of OS. WGCNA and LASSO Cox regression analyses were performed to screen for a seven-gene signature comprised of SLC18B1, RBMXL1, DOK3, HS3ST2, ATP6V0D1, CCAR1, and C1QTNF1 and associated with OS energy metabolism. Validations were performed on independent external datasets and revealed that the seven-gene signature was superior to the other models evaluated. Here, a seven-gene signature related to OS energy metabolism was constructed to forecast the outcome of this cancer. Moreover, this signature contains genes that provide potential targets for innovative and effective OS therapeutic strategies.

## Methods

### Data collection and processing

Human metabolic pathways were downloaded from the Reactome Database (http://reactome.org/) [43]. A total of 587 genes were obtained from 11 metabolic-related pathways (Table 5). RNA-seq expression and clinical follow-up data for osteosarcoma were obtained from the public database TARGET (https://ocg.cancer.gov/) [44]. There were 274 patients with clinical information and ~ 101 patients with RNA-seq data. TPM data gene length and sequencing depth (M scale RNA-seq data), were applied in this study. The following steps were performed on the 101 patients with RNA-seq data. (1) Samples lacking clinical information and/or DFS < 30 were discarded. (2) Normal tissue sample data were removed. (3) Genes with FPKM values of 0 in 50% or more of the samples were eliminated. (4) The expression profile of the genes involved in energy metabolism were retained. The gene expression profile of GSE21257 and its related clinical data were downloaded from the Gene Expression Omnibus (GEO) database (http://www.ncbi.nlm.nih.gov/geo/), and we also included the data from pre-chemotherapy biopsies of 53 patients with OS. The following steps were performed on the GSE21257 data [45]. (1) The normal tissue sample data were removed. (2) The chip probe was mapped to the human genome using the Bioconductor package in R. (3) The expression profiles of the energy metabolism-related genes were retained (Table 6).

**Table 5:** Energy metabolism-related pathways in the Reactome Database

Metabolic pathways from Reactome	PathwayID	Gene count
Biological oxidations	R-HSA-211859	216
Metabolism of carbohydrates	R-HSA-71387	290
Mitochondrial fatty acid beta-oxidation	R-HSA-77289	37
Glycogen synthesis	R-HSA-3322077	16
Glycogen metabolism	R-HSA-8982491	27
Glucose metabolism	R-HSA-70326	90
Glycogen breakdown (glycogenolysis)	R-HSA-70221	15
Glycolysis	R-HSA-70171	71
Pyruvate metabolism	R-HSA-70268	31
Pyruvate metabolism and Citric Acid (TCA) cycle	R-HSA-71406	55
Citric acid cycle (TCA cycle)	R-HSA-71403	22
Sum	11	871

**Table 6:** Clinical information for pre-processed dataset

Characteristic	TARGET training datasets (n = 76)	TARGET all datasets (n = 84)	GSE21257 (n = 53)
Age (years)			
≤ 18	59	66	35
> 18	17	18	18
Survival status			
Living	50	55	30
Dead	26	29	23
Gender			
Female	33	37	19
Male	43	47	34
Metastatic			
Metastatic	19	21	34
Non-metastatic	47	53	19

### Identification of energy metabolism molecular subtypes in OS

The OS samples were clustered with 587 energy metabolism-related genes using a non-negative matrix clustering algorithm (NMF) [46]. The “NMF” package in R was applied using the standard “burnet” for 50 iterations, setting the k cluster range from 2 to 10, determining the average contour with the common member matrix, and setting the minimum number of subclass members to ten. Based on the cophenetic correlation, dispersion, silhouette, and other indicators, the optimal cluster number was established at two [47].

### Constructing a dynamic weighted gene co-expression network

In the present study, a WGCNA co-expression algorithm was run to mine co-expressed coding genes and co-expression modules. The expression profiles for the coding genes were extracted from the TPM data in the TARGET database. The samples were clustered hierarchically to eliminate outliers. The “WGCNA” package in R was used to construct a weighted co-expression network [48], which was aligned with the scale-free network. The log(k) of the node with connection degree k was negatively correlated with the log(P(k)) of the probability of the occurrence of the node. The correlation coefficient setting was > 0.9. The co-expression modules were screened out. The gene modules were detected by average-linkage hierarchical clustering based on a topological overlap matrix (TOM)-based dissimilarity measure (1-TOM) [49]. Here, module size 80, height 0.5, deepSplit 2, and certain highly similar modules were merged. After associating the modules with clinical traits, those with the highest Pearson’s correlation coefficients were regarded as significant for the subsequent analyses.

### Gene Ontology (GO) and pathway enrichment analyses

The Gene Ontology (GO) database (http://geneontology.org/) [including biological process (BP), cell component (CC), and molecular function (MF) terms] was used to identify biological mechanisms based on high-throughput genome or transcriptome data [50]. The Kyoto Encyclopedia of Genes and Genomes (KEGG) database (http://www.kegg.jp/) [51] served to identify the systematic functions and biological relevance of candidate targets. In the present study, the “clusterProfiler” package in R (http://bioconductor.org/packages/clusterProfiler) [52] was run to conduct GO and KEGG pathway enrichment analyses of the genes in the significant modules and identify the underlying biological mechanisms.

### LASSO Cox regression and energy metabolism signature

An energy metabolism signature was constructed based on gene expression levels and associations with energy metabolism molecular subtypes. About 90% of the 84 samples from the TARGET Database were randomly selected as the training set for the signature model. The information for all samples in training set is shown in Table 6. The “survfit coxph function” package [53] in R was run to generate a univariate Cox proportional hazard regression model and expanded for the genes in significant modules and for the survival data. *P* < 0.05 was the threshold.

Least absolute shrinkage and selection operator (LASSO) estimates compression [54]. It shrinks the subset by compressing the original coefficients. It has been broadly applied to the survival analysis of high-dimensional data. In the current study, a LASSO Cox regression model was built to target the genes significantly associated with energy metabolism. A threefold cross-validation was performed for tuning parameter selection. The calculated partial deviance met the minimum criteria. A multivariate Cox survival analysis was run on significant genes. Those retained had the lowest the area under the curve (AUC) value and comprised the final gene signature for energy metabolism. To plot receiver operating characteristic (ROC) curves and compute the AUC, we ran the “pROC” package in R.

### Validation of gene signature robustness

To validate gene signature model robustness, the training set model and coefficient were applied to all genes in the TARGET Database and the external GEO dataset GSE21257 and GSE16091 [55]. The “RiskScore” was calculated for each sample based on the gene expression levels in the validation set samples (TARGET database, GSE21257, and GSE16091). The “timeROC” package in R (http://cran.r-project.org/web/packages/timeROC) [56] was run to analyze the ROC of the “RiskScore” for prognostic classification. Survival analyses of the data from the TARGET database and external GEO dataset GSE21257 and GSE16091 were performed with the “survival” package in R (http://bioconductor.org/packages/) [57]. Univariate survival was estimated by the Kaplan–Meier univariate survival method. *P* < 0.001 was considered significant.

### Gene signature model evaluation

To identify the relationship between the RiskScore of the gene signature model and the immune and matrix scores, the “estimate” package in R calculated the immune and stromal scores and the tumor purity for each sample. To identify the independence of the gene signature model in clinical applications, the relevant HR and 95% CI were evaluated via the signal factor and multivariate Cox regression in the TARGET training and validation sets and GSE21257.

### Gene set enrichment analysis (GSEA)

GSEA (http://software.broadinstitute.org/gsea/index.jsp) [58] explored the biological functions of the gene signature based on the energy metabolism status (high-risk vs. low-risk groups). The annotated gene set c2.cp.kegg.v6.0.symbols was selected as a reference. Gene size ≥ 10, *P* < 0.05, and |Enrichment Score (ES)| > 0.40 were set as the cut-off criteria.

### Comparative study of other risk models

Pertinent references were searched, and two related published risk models were selected including an eight-gene (PMID: 31333788) [45], a four-gene (PMID: 31146489) [59], a ten-gene (PMID: 31090103) [60], and a nineteen-gene (PMID: 30604867) [61] signature risk model. To make the models comparable, a multi-factor Cox regression was run and the RiskScore of the training set samples was recalculated according to the corresponding genes in the model of the present study. The ROCs of the cited (literature) models were determined. Based on the optimal threshold, the samples were divided into high- and low-risk groups and their relative impacts on OS prognosis were determined.

## Supplementary information

**Additional file 1: Table S1.** Genes in the yellowlight module.

**Additional file 2: Table S2.** Genes in the pink module.

**Additional file 3: Table S3.** GO terms for the genes in the yellowlight module.

**Additional file 4: Table S4.** KEGG terms for the genes in the yellowlight module.

**Additional file 5: Table S5.** GO terms for the genes in the pink module.

**Additional file 6: Table S6.** KEGG terms for genes in the pink module.

## Abbreviations

AUC: Area under the curve
BP: Biological process
CC: Cell component
ES: Enrichment score
GEO: Gene expression omnibus
GO: Gene ontology
GSEA: Gene set enrichment analysis
HS3ST2: Heparan sulfate-glucosamine 3-sulfotransferase 2
KEGG: Kyoto Encyclopedia of Genes and Genomes
LASSO: Least absolute shrinkage and selection operator
MF: Molecular function
NMF: Non-negative matrix clustering algorithm
OS: Osteosarcoma
OXPHOS: Oxidative phosphorylation
ROC: Receiver operating characteristic
TOM: Topological overlap matrix
WGCNA: Weighted gene co-expression network analysis
